# Retrospective case-control non-inferiority analysis of intravenous lidocaine in a colorectal surgery enhanced recovery program

**DOI:** 10.1186/s12871-017-0306-6

**Published:** 2017-01-31

**Authors:** Bhiken I. Naik, Siny Tsang, Anne Knisely, Sandeep Yerra, Marcel E. Durieux

**Affiliations:** 1 Department of Anesthesiology, University of Virginia, Charlottesville, VA USA; 2 Department of Neurosurgery, University of Virginia, Charlottesville, VA USA; 3 Department of Epidemiology, Columbia University, New York, NY USA

**Keywords:** Lidocaine, Enhanced Recovery After Surgery, Colorectal Surgery, Opioid Consumption, Pain Scores

## Abstract

**Background:**

Enhanced recovery after surgery (ERAS) programs typically utilizes multi-modal analgesia to reduce perioperative opioid consumption. Systemic lidocaine is used in several of these ERAS algorithms and has been shown to reduce opioid use after colorectal surgery. However it is unclear how much the other components of an ERAS protocol contribute to the final outcome. Using a noninferiority analysis we sought to assess the role of perioperative lidocaine in an ERAS program for colorectal surgery, using pain and opioid consumption as outcomes.

**Methods:**

We conducted a retrospective review of patients who had received intravenous lidocaine perioperatively during colorectal surgery. We matched them with patients who were managed using a multi-component ERAS protocol, which included perioperative lidocaine. We tested a joint hypothesis of noninferiority of lidocaine infusion to ERAS protocol in postoperative pain scores and opioid consumption. We assigned a noninferiority margin of 1 point (on an 11-point numerical rating scale) difference in pain and a ratio [mean (lidocaine) / mean (ERAS)] of 1.2 in opioid consumption, respectively.

**Results:**

Fifty-two patients in the lidocaine group were matched with patients in the ERAS group. With regards to opioid consumption, in the overall [1.68 (1.43–1.98)] [odds ratio (95% confidence interval)] analysis and on postoperative day (POD) 1 [2.38 (1.74–3.31)] lidocaine alone was inferior to multi-modal analgesia. On POD 2 and beyond, although the mean odds ratio for opioid consumption was 1.43 [1.43 (1.17–1.73)], the lower limit extended beyond the pre-defined cut-off of 1.2, rendering the outcome inconclusive. For pain scores lidocaine is non-inferior to ERAS [-0.17 (-1.08–0.74)] on POD 2 and beyond. Pain scores on POD 1 and in the overall cohort were inconclusive based on the noninferiority analysis.

**Conclusions:**

The addition of a multi-component ERAS protocol to intravenous lidocaine incrementally reduces opioid consumption, most evident on POD 1. For pain scores the data is inconclusive on POD 1, however on POD 2 and beyond lidocaine alone is non-inferior to an ERAS program with lidocaine. Opioid-related complications, including return of bowel function, were not different between the groups despite reduced opioid use in the ERAS group.

**Electronic supplementary material:**

The online version of this article (doi:10.1186/s12871-017-0306-6) contains supplementary material, which is available to authorized users.

## Background

Enhanced Recovery After Surgery (ERAS) protocols are evidence-based algorithms first described by Kehlet almost two decades ago [1] ERAS protocols have improved patient satisfaction and outcomes with a concomitant reduction in perioperative opioid use [2, 3].

Enhanced recovery protocols typically utilize multi-modal analgesia to reduce perioperative opioid use. Whereas the benefits of multi-modal analgesic approaches are well demonstrated, it is less clear which components are essential and which have little effect on outcome. As each component adds some degree of work, cost and potential risk, it is important to define how each component contributes to pain management.

Intravenous lidocaine has gained increasing popularity over the last decade [4–6]. Systemic lidocaine has analgesic, anti-hyperalgesic and anti-inflammatory properties, making it a virtually ideal agent for pain management during the perioperative period [7]. The short- and long-term benefits of lidocaine have been demonstrated across a variety of surgical procedures including colorectal, breast and spine surgery [6, 8, 9]. Several meta-analyses have reported that intravenous lidocaine during abdominal surgery decreases postoperative pain severity, reduces opioid consumption and nausea and vomiting, improves gastrointestinal function and shortens length of hospital stay [4, 5]. Using a noninferiority analysis we recently reported that after major abdominal surgery patients who received intravenous lidocaine had clinically similar pain scores on postoperative day (POD) 2 and beyond compared with patients receiving epidural analgesia [10].

Given these effects, it can be hypothesized that intravenous lidocaine provides the majority of the analgesic benefits of any ERAS protocol, even if the protocol contains many additional interventions. If so, those other components could be eliminated without affecting outcome, which would likely reduce cost and improves safety. At our institution we utilized perioperative lidocaine with opioid-based analgesia for colorectal surgery, and then implemented an ERAS program that included perioperative lidocaine but added multi-modal analgesia, opioid restriction and fluid and activity interventions. This practice change allowed a comparison between the two approaches as to pain scores, opioid consumption and side effects.

We hypothesized that in colorectal surgery perioperative systemic lidocaine with opioid analgesia is non-inferior to a full ERAS protocol with regards to the endpoints of postoperative pain scores and opioid consumption.

## Methods

This study was initially performed as a quality improvement project with approval from our local departmental quality improvement committee. Subsequent approval for publication of the data was obtained from the University of Virginia institutional review board (HSR #10712) and the need to obtain consent was waived. The study period was from January 2013 to June 2015 and included all patients who underwent colorectal surgery. The two cohorts identified were patients who received perioperative lidocaine with opioid-based postoperative analgesia (LIDO group) and those who were part of an ERAS program that included multi-modal analgesia and perioperative lidocaine, as well as restriction of intravenous fluids and encouragement of oral fluid intake and ambulation (ERAS group). Patients were matched by age (within 5 years), gender and chronic opioid use (>1 month).

As described in our previous study, we assigned a noninferiority margin of 1-point (on an 11-point numerical rating scale) difference in pain and a ratio [mean (LIDO) / mean (ERAS)] of 1.2 in opioid consumption [10, 11]. Noninferiority is established when the lower bound of the 95% confidence interval (CI) does not cross the inferiority margin, whereas the comparison is rendered inconclusive if the lower bound 95% CI does cross the inferiority margin [12]. We wished to know if lidocaine alone was no less effective than an ERAS program; therefore a non-inferiority analysis, as opposed to a superiority analysis was conducted.

The primary outcome was patient-reported pain scores at rest and opioid consumption for the first four postoperative days or discharge if earlier. Secondary outcomes include hypotension (defined by any blood pressure requiring adjusting/holding the pain regimen, additional fluid administration or administration of inotropes/vasopressors), patient-reported nausea and documented vomiting, pruritus, urinary retention requiring catheterization, duration of indwelling urinary catheterization, time to first ambulation, time to first bowel movement and duration of hospital stay. The regional anesthesia team monitored the lidocaine infusion and collected data on patient satisfaction and mental status.

### Anesthetic and analgesic regimen

The anesthetic regimen in both groups included general anesthesia with endotracheal intubation. The typical induction agent was propofol with muscle relaxation achieved with either succinylcholine or rocuronium. Anesthesia was maintained with either sevoflurane or desflurane. If reversal of neuromuscular blockade was required, neostigmine and glycopyrrolate were administered.

#### LIDO group

In the LIDO group, intraoperative opioid administration was at the discretion of the anesthesiology provider. A lidocaine infusion was started following induction of general anesthesia at a rate of 2–3 mg/min, based on previous reported dosing regimens [13, 14]. Prior to transfer to the recovery room the infusion was decreased to 0.5–1 mg/min. The infusion was continued between 0.5 and 1 mg/min for 2–5 days postoperatively.

Postoperatively, hydromorphone patient-controlled analgesia (PCA) was commenced at a dose of 0.1 to 0.2 mg hydromorphone per bolus, and opioid-tolerant patients received 0.3 to 0.4 mg hydromorphone per bolus; both groups had an 8-min lockout. Patients who did not receive a PCA were given 0.5 to 1.0 mg intravenous hydromorphone every hour as needed. Acetaminophen was administered intravenously initially with a total dose that did not exceed 4 gram/day. Acetaminophen was changed to an oral formulation when oral intake resumed. Oxycodone was started when oral intake permitted to facilitate transitioning off the PCA. At this time the lidocaine infusion was typically discontinued.

#### ERAS group

In the ERAS group, prior to surgery, patients received an oral regimen of celecoxib 200 mg (not given to patient with coronary artery disease), acetaminophen 975 mg and gabapentin 600 mg (Table 1).

**Table 1:** Perioperative analgesia regimen in the standard therapy-perioperative lidocaine and enhanced recovery multi-modal analgesia-perioperative lidocaine group

Perioperative Analgesia Regimen		
	Standard Therapy-Perioperative Lidocaine	ER Multi-Modal Analgesia-Perioperative Lidocaine
Preoperative	1. None	1. Celecoxib 200 mg PO^c^ 2. Acetaminophen 975 mg PO 3. Gabapentin 600 mg PO
Intraoperative	1. Intravenous lidocaine 2–3 mg/min 2. Intravenous Opioids^a^ 3. Intravenous Ketamine^a^	1. Intravenous lidocaine 2–3 mg/min 2. Single intrathecal dose of preservative free morphine (100 μg) 3. Ketamine 0.5 mg/kg IV at induction followed by an infusion at 0.6 mg/kg/h (stopped 45-min before closure for laparoscopic cases and decreased to 0.3 mg/kg/h in open cases until completion of the case 4. Magnesium 30 mg/kg intravenously at induction 5. Dexamethasone 4 mg at induction 6. Intravenous opioids^b^
Postoperatively	1. Intravenous lidocaine 0.5–1 mg/min 2. Hydromorphone PCA and oral oxycodone 3. Acetaminophen	1. Intravenous lidocaine 0.5–1 mg/min 2. Celecoxib 100 mg twice daily 3. No opioid PCA 4. Oral oxycodone 5. Acetaminophen

*PO*-per oral, *μg*-microgram, *mg*-milligram

^a^Discretion of anesthesiology provider. ^b^After approval of attending anesthesiologist.^c^Not administered to patients with coronary artery disease

The components of the intraoperative multi-modal analgesia included: (1) Prior to induction of anesthesia, a single intrathecal dose of preservative free morphine (100 mcg), (2) ketamine 0.5 mg/kg IV at induction followed by an infusion at 0.6 mg/kg/h (stopped 45-min before closure for laparoscopic cases and decreased to 0.3 mg/kg/h in open cases until completion of the case (3) magnesium 30 mg/kg intravenously at induction, (4) dexamethasone 4 mg at induction (Table 1).

An intravenous lidocaine infusion was commenced as in the LIDO group for the perioperative period. Intraoperative intravenous opioid administration was only administered following approval of the attending anesthesiologist.

Postoperatively 1 g intravenous acetaminophen was administered every 6 h after initial dose and every 6 h subsequently. Oral oxycodone 5 mg, 10 mg and 15 mg every 4 h as required for mild, moderate and severe pain respectively was prescribed. No PCA was utilized. Celecoxib 100 mg was administered twice daily in patients without coronary artery disease.

### Data collection and synthesis

Perioperative data was collected from the anesthesia information system and electronic health record. All opioids used during the intraoperative and postoperative period were converted to morphine equivalents for analysis [15]. Intrathecal morphine was converted to oral morphine by a ratio of 1: 100. A 11-point visual analog scale (VAS) was used to rate postoperative pain. Hypotension was defined as any blood pressure that required administration of fluids, inotropes, or vasopressors or withholding pain medication. Postoperative nausea was identified as any patient requiring treatment. Time to ambulation, first bowel movement or passage of flatus, removal of indwelling urinary catheter and discharge from hospital were calculated from the time the patient left the operating room. Patient satisfaction was measured using a binary variable (yes/no). Patients were asked regularly about peri-oral numbness/tingling, dizziness, tinnitus, diplopia, seizures, arrhythmia, extremity numbness and muscle twitching to assess for lidocaine toxicity, as is standard for our practice. Postoperative mental status was assessed using the following scale: awake/alert, confused, somnolent, arouses with simulation, difficult to arouse, or unresponsive.

### Power analysis

As we did not foresee the use of the data for the current study at the time of data collection, an *a priori* power analysis was not conducted. The size of the confidence interval provides an indication of the likelihood of the real effect size being zero or very small. For our non-inferiority study, the upper limit of the confidence interval is an estimation of the maximum effect size supported by the current data.

We feel it is inappropriate to perform post-hoc power calculations in this setting, because the calculated power (i.e., “observed” power obtained from the model estimates) is a function of the *p*-values of the model estimates, meaning that post-hoc power analysis does not provide additional information to the results [16]. In addition, when there is a non-significant finding (such as the ones in the current study), a higher post-hoc power provides stronger evidence against the null hypothesis [17].

### Statistical analysis

Normality of data was established using the Shapiro-Wilk test. Normally distributed data are reported as mean and SD while non-normally distributed data are reported as median and 25th-75th interquartile range.

The two primary outcomes (postoperative pain and opioid consumption) were assessed postoperatively in the recovery room, and every 12 h for up to 4 days. Mixed-effects models were used to examine the changes in the primary outcomes over time, taking into account the within-individual correlations between assessments.

The mixed-effects model found to be best fit the changes in the overall postoperative pain scores is:

$$ {Y}_{i j}={\upbeta}_0+{\upbeta}_1{x}_{1 ij}+{\upbeta}_2{x}_{2 ij} + {{\mathrm{b}}_i}_0 + {{\mathrm{b}}_i}_1{z}_{1 ij}+{\upvarepsilon}_{i j} $$

where *Y*_*ij*_ is the postoperative pain score for observation *j* in patient *i*; *x*_1*ij*_ refers to the linear time (number of days), and *x*_2*ij*_ refers to the analgesia group (ERAS vs. LIDO) fixed effects for observation *j* in patient *i*; β_0_ is the random intercept for patient *i*, *z*_1*ij*_ is the random slope for time; and ε_*ij*_ is the error for observation *j* in patient *i*. This model indicates that the change in postoperative pain scores follows a linear trend over time, with between-patient heterogeneity in initial postoperative pain scores (in recovery room) and the rate of changes in pain scores across time. The same model was fitted to the changes in postoperative pain scores POD1 only, and POD2 to POD4.

The mixed-effects model found to be best fit the changes in the overall postoperative morphine consumption is:

$$ {Y}_{i j}={\upbeta}_0+{\upbeta}_1{x}_{1 ij}+{\upbeta}_2{x}_{2 ij}+{\upbeta}_3{x}_{3 ij}+{{\mathrm{b}}_i}_0+{{\mathrm{b}}_i}_1{z}_{1 ij}+{\upvarepsilon}_{i j} $$

where *Y*_*ij*_ is the postoperative morphine consumption for observation *j* in patient *i*; *x*_1*ij*_ refers to the linear time (number of days), *x*_2*ij*_ refers to the quadratic (non-linear) time, and *x*_3*ij*_ refers to the analgesia group (ERAS vs. LIDO) fixed effects for observation *j* in patient *i*; β_0_ is the random intercept for patient *i*, *z*_1*ij*_ is the random slope for time; and ε_*ij*_ is the error for observation *j* in patient *i*. This model indicates that the change in morphine consumption follows a non-linear trend over time, with between-patient heterogeneity in initial postoperative pain scores (in recovery room) and the rate of changes in pain scores across time. The same model was fitted to the changes in postoperative morphine consumption POD1 only. However, the model without the quadratic time fixed effects was fitted to the changes in postoperative morphine consumption on POD2 to POD4, meaning that the changes in postoperative morphine consumption POD2 to POD4 follows a linear trend over time.

Fisher’s exact test or Mann-Whitney *U* test was used to compare the incidences of each secondary outcome. All statistical analyses were conducted using the statistical program R version 3.2 (R Core Team (2015). R: A language and environment for statistical computing. R Foundation for Statistical Computing, Vienna, Austria. URL https://www.R-project.org/).

## Results

Fifty-two patients in the LIDO group were matched with 52 patients in the ERAS group. Perioperative variables are shown in Table 2. No significant differences in demographic and preoperative opioid consumption were noted. Total intraoperative opioid use was similar between the groups (LIDO: 18.8 [10.6-27.5] mg vs. ERAS: 15 [7.50–25] mg, *p* = 0.14), however significantly more intravenous opioids were administered in the lidocaine group while there was more intrathecal opioid use in the ERAS group. This was primarily related to the ERAS protocol limiting intravenous administration of opioids. All patients in the ERAS group received oral acetaminophen, gabapentin and celecoxib prior to surgery. No patient in the standard group received oral acetaminophen, gabapentin and celecoxib prior to surgery. Total lidocaine dose was higher in the LIDO group (LIDO: 2888 [2188-4322] mg versus ERAS: 1557 [959-1992] mg, *p* = 0.0001), primarily due to the earlier discharge in the ERAS group. An additional supplementary file titled ‘Database’ reports the raw data in more detail [see Additional file 1].

**Table 2:** Perioperative data in the standard therapy-perioperative lidocaine and enhanced recovery multi-modal analgesia-perioperative lidocaine group

	Standard Therapy-Perioperative Lidocaine	ER Multi-Modal Analgesia-Perioperative Lidocaine	*P*-value
Preoperative			
Age (years)^a^	52.8 (14.6)	53.5 (13)	0.79
Gender (% men)^b^	50	50	1
BMI (kg/m2)^c^	26.11 [22.9-29.8]	26.7 [23.6-31]	0.47
Chronic pain (%)^b^	44	44	1
Preoperative morphine equivalent (mg)^c^	0 [0-23.3]	0 [0-10]	0.17
Intraoperative			
Laparoscopic Procedures n (%)	12 (23%)	27 (52%)	0.004
Total Intraoperative morphine equivalent (mg)^c^	18.8 [10.6-27.5]	15 [7.50-25]	0.14
Intrathecal Morphine (mg)	0 [0-0]	0.15 [0.1-0.25]	0.0001
Intravenous Fentanyl (μg)	150 [100-250]	0 [0-0]	0.0001
Intravenous Hydromorphone (mg)	1 [0.4-2]	0 [0-0]	0.0001
Ketamine (mg)	0 [0-60.7]	102 [63.8-150.2]	0.0001
Magnesium (mg)	0 [0-0]	2000 [2000-2500]	0.0001
Lidocaine (mg)	494 [379-615]	592 [388-900]	0.13
Postoperative			
Lidocaine (mg)	2888 [2188-4322]	1557 [959-1992]	0.0001

^a^Presented as mean (SD), *P* value from simple *t* test

^b^Presented as frequency, *P* value from *χ*2 or Fisher exact test

^c^Presented as median and (IQR), *P* value from Mann-Whitney *U* test

### Primary outcome

**Fig. 1:**
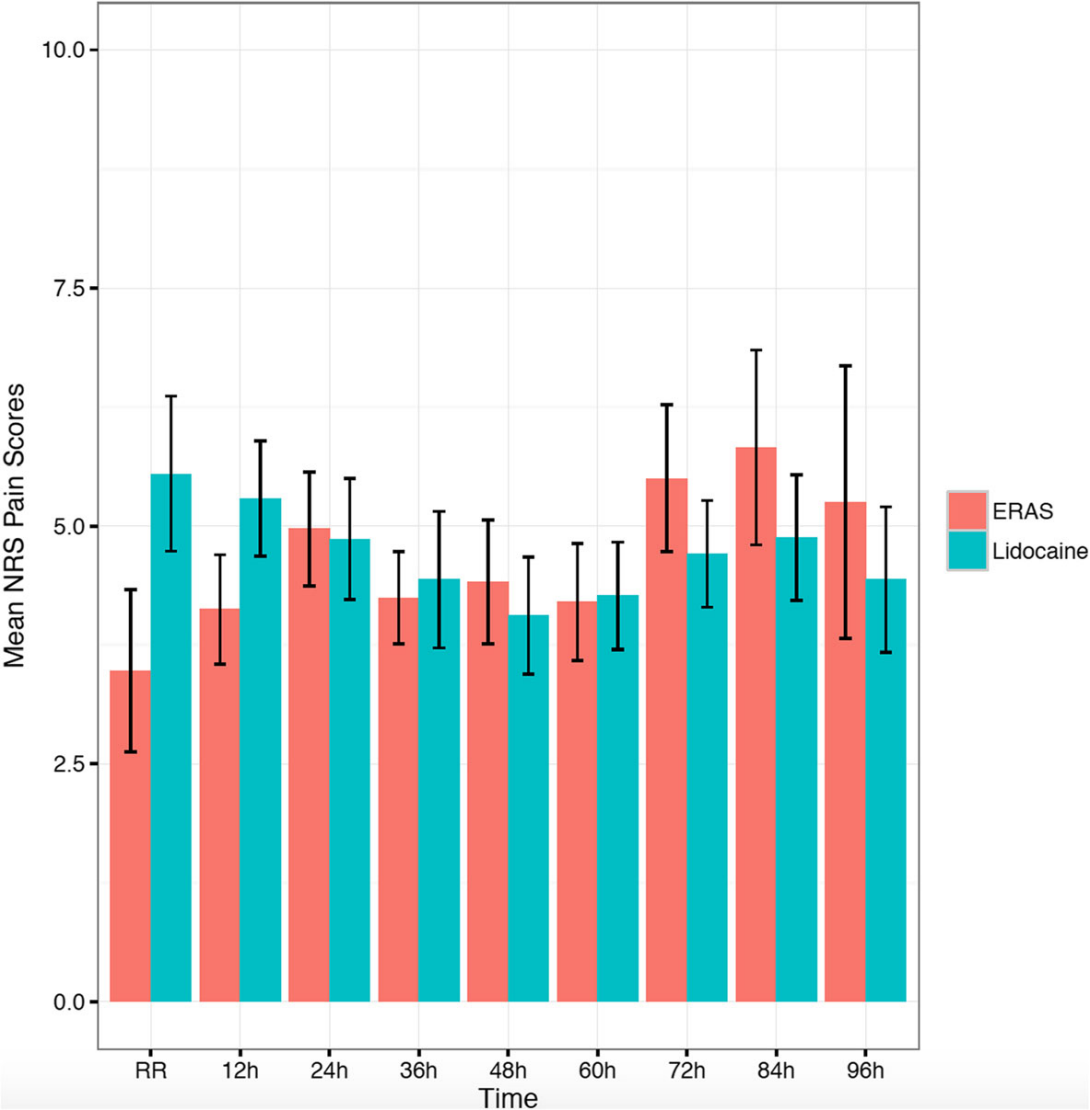
Mean and standard error of postoperative NRS pain scores. NRS: Numerical Rating Scale, ERAS: Enhanced Recovery After Surgery

As previously described, the non-inferiority margin for pain was defined *a priori* as a 1-point difference between the two groups. Mean pain scores are represented in Fig. 1. Our data demonstrate that for the overall study period the upper limit 95% confidence interval extends beyond the 1-point difference (Table 3, Fig. 3), indicating one can not conclude from the data that lidocaine is inferior to ERAS group. The pain score for POD 1 is similar, with the mean difference and 95% confidence interval 1.16 (0.15–2.18); thus, although the difference falls within the non-inferiority range, the upper limit CI extends beyond the 1-point difference (Table 3, Fig. 3a). For POD 2 and beyond lidocaine is non-inferior to ERAS [-0.17 (-1.08–0.74)].

**Table 3:** Difference in NRS pain scores and opioid consumption

Difference in NRS Pain Scores and Opioid Consumption					
Difference in NRS Pain Scores					
	Standard Therapy-Perioperative Lidocaine	ER Multi-Modal Analgesia-Perioperative Lidocaine	Mean Difference (ST − ER) (95% CI)	Δ	*p*-value
Overall	4.7 (2)	4.5 (2)	0.43 (-0.46-1.31)	1	0.1
	4.5 [3.0, 6.0]	4.7 [2.7, 6.0]			
Day 1	5.23 (2.12)	4.17 (2.10)	1.16 (0.15-2.18)	1	0.62
	5.11 [3.65, 6.76]	4.02 [2.60, 5.88]			
Day 2 and beyond	4.44 (2.06)	4.70 (2.04)	-0.17 (-1.08-0.74)	1	0.006
	4.49 [2.66, 6.00]	4.77 [3.40, 6.22]			
Difference in Opioid Consumption					
	Standard Therapy-Perioperative Lidocaine	ER Multi-Modal Analgesia-Perioperative Lidocaine	Mean ST/Mean ER Opioid Consumption (95% CI)		
Overall	52.54 (64.45)	31.22 (36.62)	1.68 (1.43-1.98)	1.2	<0.0001
	30.20 [17.32, 54.94]	22.91 [9.77, 40.39]			
Day 1	43.77 (54.02)	18.43 (28.83)	2.38 (1.74-3.31)	1.2	<0.0001
	25.18 [13.93, 46.54]	9.95 [1.95, 23.78]			
Day 2 and beyond	57.03 (72.42)	39.93 (40.58)	1.43 (1.17-1.73)	1.2	0.8
	27.46 [16.15, 71.43]	28.42 [15.98, 47.68]			

Data presented as mean (SD) and median [IQR]

**Fig. 2:**
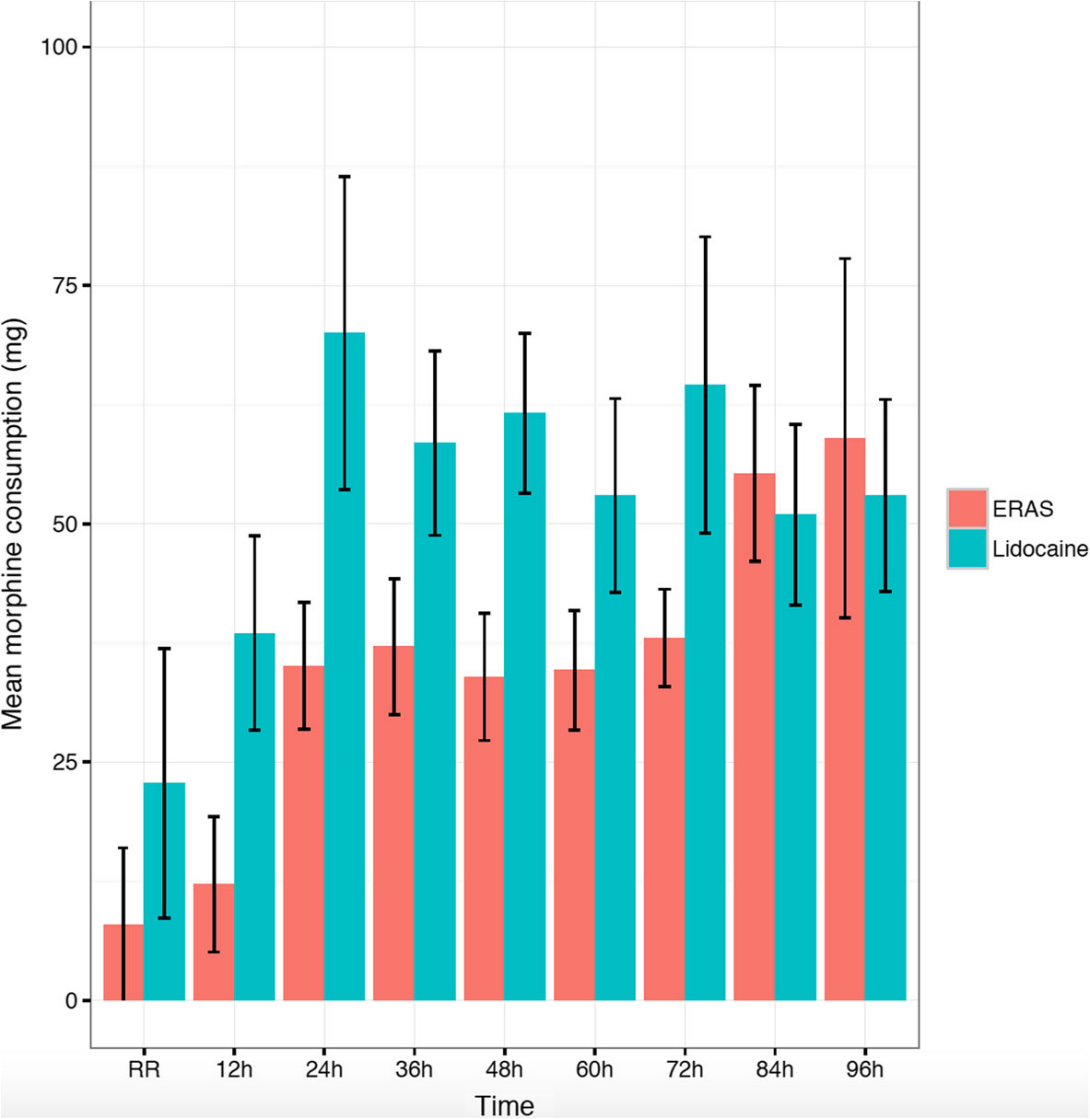
Mean and standard error of opioid (morphine equivalent) consumption. ERAS: Enhanced Recovery After Surgery

**Fig. 3:**
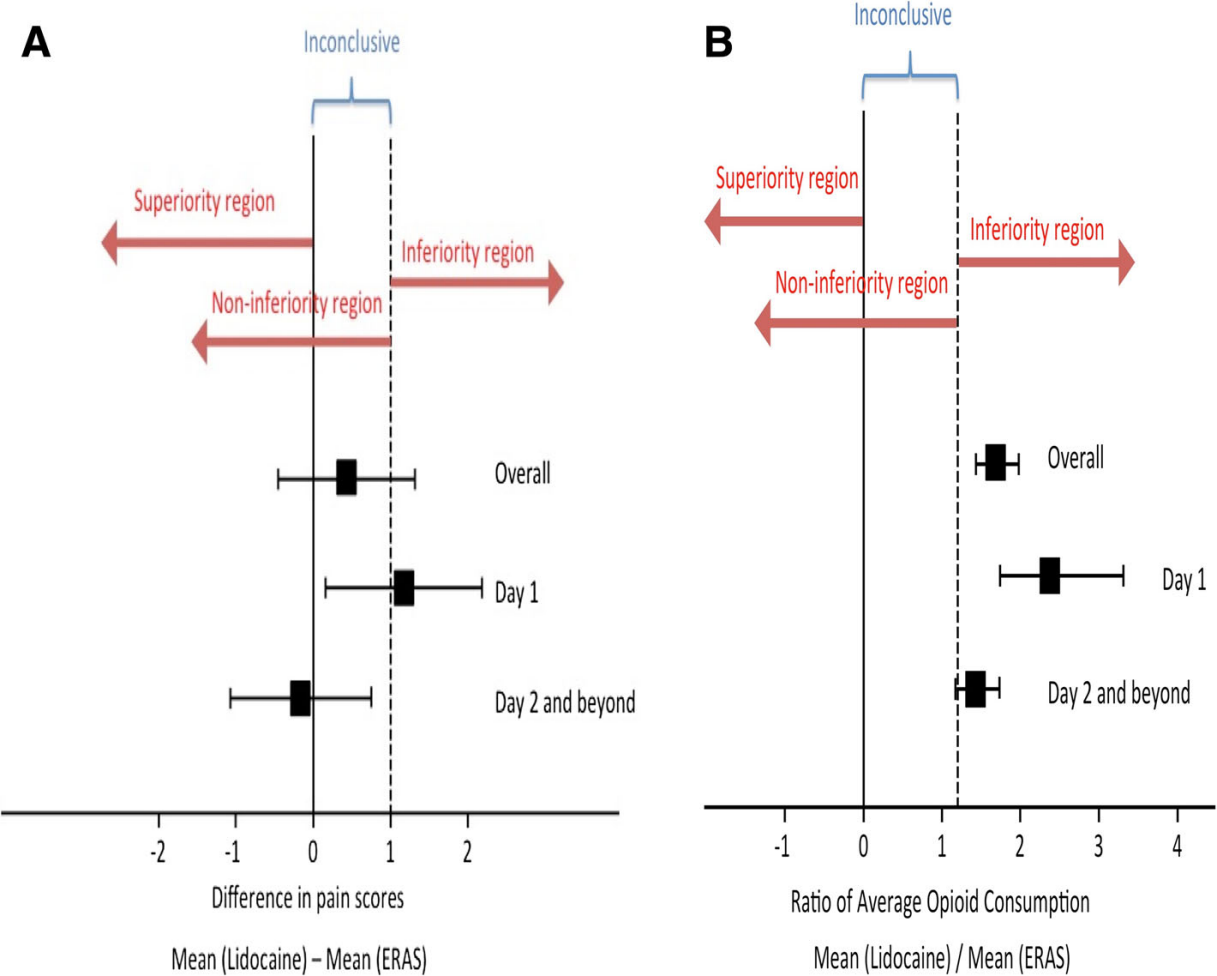
Noninferiority margins for pain scores (**a**) and opioid consumption (**b**). Squares represent the mean and the whiskers represent the 95% confidence interval. ERAS: Enhanced Recovery After Surgery

For opioid consumption, the non-inferiority limit was defined *a priori* as a ratio of mean lidocaine / mean ERAS of 1.2. Mean opioid use (morphine equivalents) is demonstrated in Fig. 2. In the overall and POD 1 group lidocaine was inferior to ERAS for opioid consumption (Table 3, Fig. 3b). For POD 2 and beyond, although the mean ratio was 1.43, the lower limit extended beyond the pre-defined cut-off, rendering the outcome inconclusive.

### Secondary outcome

Secondary outcomes are listed in Table 4. There was no difference noted in the incidence of hypotension and nausea/vomiting between the groups. There was an increased incidence of pruritus present on POD 2 in the LIDO group, however this was not evident on POD 3 and 4. In the LIDO group a higher percentage of patients with either confusion, somnolence, difficulty with arousal or unresponsive was evident on POD 2 (LIDO: 11.54% vs. ERAS: 0%, *p* = 0.03).

**Table 4:** Secondary outcomes

	Standard Therapy-Perioperative Lidocaine	ER Multi-Modal Analgesia-Perioperative Lidocaine	*P* value
Hypotension^a^			
POD 1	0 (52); 0%	1 (52); 1.92%	1
POD 2	0 (52); 0%	0 (49); 0%	-
POD 3	0 (40); 0%	0 (10); 0%	-
POD 4	1 (23); 4.35%	0 (5); 0%	1
Nausea and Vomiting^a^			
POD 1	11 (52); 21.15%	6 (52); 11.54%	0.29
POD 2	16 (52); 30.77%	7 (49); 14.29%	0.06
POD 3	9 (40); 22.50%	5 (10); 50%	0.12
POD 4	8 (23); 34.78%	2 (5); 40%	1
Pruritis^a^			
POD 1	5 (52); 9.62%	2 (52); 0.04%	0.44
POD 2	13 (52); 25%	2 (49); 4.08%	0.004
POD 3	6 (40); 15%	1 (10); 10%	1
POD 4	2 (23); 8.70%	0 (5); 0%	1
Mental status: not awake or alert^a^			
POD 1	4 (52); 7.69%	2 (52); 0.04%	0.68
POD 2	6 (52); 11.54%	0 (49); 0%	0.03
POD 3	3 (40); 7.50%	0 (10); 0%	1
POD 4	0 (23); 0%	0 (5); 0%	-
Urinary retention^a^			
POD 1	1 (52); 1.92%	6 (52); 0.12%	0.11
POD 2	1 (52); 1.92%	3 (49); 6.12%	0.35
POD 3	31 (40); 77.50%	6 (10); 60%	0.42
POD 4	0 (22); 0%	1 (5); 20%	0.19
Patient Satisfaction: yes^a^			
POD 1	34 (52); 65.38%	38 (52); 73.08%	0.52
POD 2	42 (52); 80.77%	40 (49); 81.63%	1
POD 3	31 (40); 77.50%	6 (10); 60%	0.42
POD 4	19 (23); 82.61%	1 (5); 20%	0.01
Time to ambulation (hr)^bc^	44.50 [22, 65.50]	20 [13.50, 25.75]	< .001
Duration of urine catheter (hr)^bc^	43.50 [25.75, 67.50]	27 [20, 42]	0.006
Time to bowel movement (hr)^bc^	40 [18, 71.50]	41.25 [28.38, 51]	0.63
Duration hospital stay (hr)^bc^	146 [96.75, 288]	72.25 [66.75, 114.12]	< .001

^a^Presented as frequency, *P* value from *χ*2 or Fisher exact tests

^b^Presented as median and (IQR), *P* value from Mann-Whitney *U* test

^c^All these measurements calculated from the time the patient left the operating room

Time to ambulation, duration of bladder catheterization and duration of hospital stay were significantly reduced in the ERAS group (Table 4). No difference in the time to first bowel movement was noted between the groups. Total postoperative lidocaine dose was significantly higher in LIDO group.

## Discussion

Our study demonstrates incremental benefit of adding the components of a full ERAS protocol (including multi-modal analgesics) to perioperative lidocaine for reducing opioid requirements after colorectal surgery. The opioid-sparing effects are most evident on POD 1 and extend into POD 2 and beyond even though it does not reach the statistical significance level defined *a prior*.

Although pain scores tended to be higher in the LIDO group, the non-inferiority analysis is inconclusive on POD 1 and in the overall cohort. However, pain scores demonstrate noninferiority in the standard care-perioperative lidocaine group on POD 2 and beyond. It is important to note these findings were present despite there being significantly more open (and presumably more painful) procedures in the LIDO group (23% vs. 52%, *p* = 0.004).

The reduced time to ambulation, duration of urinary catheterization and hospital stay noted in the ERAS group are related to standardized goals for early ambulation and removal of urinary catheters.

ERAS programs are designed to reduce opioid consumption, opioid-related complications (nausea, ileus, etc.) and to encourage early mobilization. It is important to note that the early ambulation noted in the ERAS group (ERAS: 20 [13.50, 25.75] hrs vs. LIDO: 44.5 [22, 65.5] hrs) potentially may have contributed to increase pain and opioid consumption, which was obviated by intraoperative multi-modal analgesia (Table 1). Based on our results the addition of this multi-modal analgesic regimen to perioperative lidocaine is associated with a reduction in opioid consumption most evident on POD 1, but interestingly not with a decrease in opioid-related side effects.

The combination of intraoperative intrathecal morphine, ketamine and magnesium likely contributed to the significant reduction in opioid requirements noted on POD 1 (LIDO: 25.18 [13.93, 46.54] mg vs. ERAS: 9.95 [1.95, 23.78] mg). This effect is less striking on POD 2 and beyond (LIDO: 27.46 [16.15, 71.43] mg vs. ERAS: 28.42 [15.98, 47.68] mg) and probably reflects the waning effect of neuraxial morphine and N-methyl-D-aspartate (NMDA) antagonist.

A single dose of intrathecal morphine prior to surgery reduces postoperative enteral and parenteral opioid requirements after abdominal surgery. The duration of opioid-sparing depends on the type of opioid and the dose administered. Reported benefits of a single dose of intrathecal morphine are evident up to 24 h after administration [18, 19]. These findings are consistent with our data, which demonstrates significant opioid sparing effect in the ERAS group on POD 1. Complications of intrathecal morphine such as pruritus, respiratory depression and nausea/vomiting are not evident in our study, with no clinically significant morbidity noted in the group that received intrathecal morphine (Table 4). Furthermore, alterations in mental status, which would be a surrogate marker of significant respiratory depression, were not different between the groups (LIDO: 7.69% vs. ERAS: 0.04%, *p* = 0.11] on POD 1.

The inclusion of ketamine also likely played a role in reducing postoperative opioid use. In abdominal surgery sub-anesthetic doses of ketamine modulate opioid-induced hyperalgesia via the NMDA receptor reducing postoperative pain and opioid consumption [20, 21]. The anti-nociceptive benefits of low-dose ketamine are evident up to 24 h after surgery. Anti-nociceptive effects mediated via NMDA antagonism are more pronounced when a second NMDA antagonist is added to ketamine. Magnesium, an endogenous voltage-dependent NMDA receptor-channel blocker, demonstrates synergism with ketamine. In a rat model of acute nociception, the combination of magnesium and ketamine was more effective in reducing pain than either drug alone [22, 23].

Although our data demonstrates reduction in opioid use in the ERAS group, opioid-related complications were not significantly different between the two groups. Somewhat surprisingly, return of bowel function, a major side effect of opioids, was not different (LIDO: 40 [18, 71.50] h vs. ERAS 41.3[28.38, 51] h, *p* = 0.63) between the two groups.

The opioid consumption and pain scores profile reported here are similar to findings presented in our previous study comparing lidocaine to epidural analgesia for major abdominal surgery [10]. In that study intravenous lidocaine was inferior to epidural for opioid consumption (overall, POD 1 and POD 2 beyond) with pain scores demonstrating non-inferiority on POD 2 and beyond.

There are several limitations of this study. In the LIDO group no pre-defined algorithm for opioid administration was utilized with opioid administration solely determined by the clinical team managing the patient. This variability may have influenced our results. We had a small study size, which reduces the chance of detecting a true effect or reduces the likelihood that a statistically significant result reflects a true effect. This was a single-center study investigating an institutional specific ERAS program, therefore our results may not be translatable to other institutions using different ERAS programs. Finally this was retrospective chart review and the possibility of misclassification bias or incorrectly coded data in the electronic medical record cannot be excluded.

As reported in our previous study, there are no standardized criteria for non-inferiority analysis [10]. We utilized a noninferiority margin of 1-point (on an 11-point numerical rating scale) difference in pain and a ratio [mean (lidocaine)/ mean (ERAS)] of 1.2 in opioid consumption for consistent reporting between our studies. Changing the noninferiority margin can potentially alter the outcomes of our results. Finally, we acknowledge the limitation of the lack of an *a priori* power analysis in the current study; however, power analysis for future studies can be performed with the current data.

## Conclusions

In conclusion, our data suggest that ERAS protocols that include intraoperative intrathecal opioids and non-opioid analgesics have a distinct opioid-sparing effect within the first 24 h compared to lidocaine alone. We suggest that a combination of intraoperative protocolized pain management with postoperative continuation of intravenous lidocaine has distinct benefits on pain scores and opioid use for colorectal surgery.

## Additional file

Additional file 1: Database. Raw, de-identified data in an excel spreadsheet. (XLSX 93 kb)

## Abbreviations

CI: Confidence interval
ERAS: Enhanced Recovery After Surgery
LIDO group: Perioperative lidocaine with opioid-based postoperative analgesia
NMDA: N-methyl-D-aspartate
PCA: Patient-controlled analgesia
POD: Postoperative day
VAS: Visual analog scale
